# Peripheral Contrast Reduction Optically Induced by Scattering Lenses Thickens Peripheral Choroid

**DOI:** 10.1167/tvst.13.10.32

**Published:** 2024-10-22

**Authors:** Antonia Roth, Katharina Breher, Sandra Gisbert, Augusto Arias, Susanna Pearline Clement, Siegfried Wahl

**Affiliations:** 1 Institute for Ophthalmic Research, University of Tübingen, Tübingen, Germany; 2 Carl Zeiss Vision International GmbH, Aalen, Germany

**Keywords:** contrast reduction, scattering, choroidal thickness, axial length, visual acuity

## Abstract

**Purpose:**

The mechanisms underlying a myopia control strategy using scattering lenses are unclear. Therefore, this study investigates the short-term effects of scatter lenses on central and peripheral choroidal thickness and axial length, which serve as a biomarker in myopia progression research.

**Methods:**

In total, 23 participants underwent a 60-minute lens wear phase each to five lens conditions: medium peripheral scattering, high peripheral scattering, medium full-field scattering, high full-field scattering and control (clear lens). Central and peripheral choroidal thickness, foveal axial length, and central visual acuity were measured before and after each lens wear condition.

**Results:**

Peripheral choroidal thickening was found after the lens wear phase of the medium peripheral scattering condition (+3.91 ± 5.37 µm, *P* = 0*.*04), revealing a significant difference to the control lens condition (*P* = 0.004), most pronounced in the superior peripheral retina (+1.95 ± 10.74 µm, *P* = 0.02). In the central retina, significant choroidal thickening was only found in the nasal part after exposure to medium full-field scattering (+3.91 ± 11.72 µm) compared to the control condition (*P* = 0.001). High peripheral and full-field scattering conditions did not significantly affect central or peripheral choroidal thickness. Visual acuity was significantly reduced in the full-field scattering conditions compared to control and peripheral scattering lenses, with no improvement after 60-minute lens wear. Axial length did not differ significantly after 60-minute exposure to any scattering lens condition or when compared to the control lens.

**Conclusions:**

The results indicate a local retinal contrast detection mechanism signals the choroid to thicken peripherally after adaptation to medium peripheral scattering but not high peripheral scattering or full-field scattering at all, while central thickening was only significant nasally after exposure to medium full-field scattering. This emphasizes the importance of the peripheral retina and the level of contrast reduction in the context of myopia research.

**Translational Relevance:**

This finding gives insight into the mechanism behind the myopia control strategy inducing peripheral scattering.

## Introduction

Retinal image quality and image contrast processing are suggested to regulate eye growth.^1^^–^^3^ The deterioration of the retinal image quality leads to a disruption of the normal growth patterns of the eye, resulting in extensive ocular elongation.^2^ This abnormality in eye growth can cause the development of myopic refractive error.^1^^,^^2^ Myopia is a growing public health concern worldwide due to its increasing prevalence.^4^^,^^5^ The development of myopia is influenced by a combination of environmental and genetic factors.^6^ Exposure to higher illumination levels, wider spectral composition, higher contrast levels, and higher spatial frequencies are thought to underlie the protective effect of outdoor activity against myopia development.^7^^,^^8^ Conversely, near-work is thought to contribute to myopia development, potentially due to accommodative demand, reduced illumination, and lower contrast levels that accompany near-work.^9^ Genome-wide association studies have identified multiple genes associated with myopia, shedding light on the molecular pathways underlying this condition.^10^ Moreover, the robust correlation between myopia and parental history,^11^ along with consistent findings from heritability studies,^12^ shows that over half of the variability in refractive errors among populations is influenced by genetic factors.

The interaction of genetics and lifestyle factors is thought to trigger excessive ocular elongation, as well as myopia development and progression. This excessive elongation is oftentimes accompanied by degenerative changes in the retina, choroid, and sclera,^13^ which can lead to pathologic conditions that may irreversibly reduce vision such as maculopathy, retinal detachment, choroidal neovascularization, cataracts, and glaucoma.^13^^,^^14^

Given the severe risks associated with pathologic myopia, slowing down the rate of myopia progression during childhood has become a key goal. Numerous approaches have been described in the literature to slow the progression of myopia, such as pharmaceutical interventions (e.g., atropine eye drops),^15^^,^^16^ light therapy,^17^^–^^19^ and optical strategies, which are applied as spectacle lenses, contact lenses, or orthokeratology.^20^^,^^21^ Current optical approaches are designed to induce peripheral defocus (such as defocus incorporated multiple segments^22^ and highly aspherical lenslet lenses),^23^ diminish the lag of accommodation (e.g., progressive addition lenses),^24^ and decrease retinal image contrast as utilized by diffusion optics technology lenses.^25^ Several studies have remarked on the importance of the peripheral retina as a crucial area, in the strategy to control myopia.^26^^–^^29^

Previous literature shows that a mutation affecting mid- or long-wavelength cone opsins (OPN1MW or OPN1LW, respectively) results in a deficiency or an absence of opsin function in all cones harboring the mutation. This is thought to produce abnormally high contrast signaling between the affected and nonaffected cones, which may be a signal for eye growth.^25^^,^^30^ It has been suggested that through a reduction in retinal image contrast, the ON- and OFF-bipolar cell channel pathways, which are responsible for processing image contrast, may reduce their firing rate and the stimulus to eye growth, effectively slowing ocular elongation.^31^ The reduced contrast response in the ON and OFF channel pathways is suggested to slow axial elongation. The diffusion optics technology lens design is characterized by integrating light-scattering elements on the peripheral spectacle lens surface while maintaining clear vision with a central aperture of 5 mm.^25^^,^^32^ The concept aims to reduce peripheral retinal image contrast and thus abnormal high contrast signaling.^25^ A clinical trial study reported reduced axial elongation of −0.13 mm in myopic children when wearing diffusion optic technology spectacle lenses compared to myopes wearing single-vision lenses—after 36 months of lens wear. ^25^^,^^33^ Numerous studies have been conducted in animal models to elucidate the mechanisms that regulate eye growth, providing insights into human myopia. It is widely demonstrated that by covering eyes with positive or negative lenses, the eye compensates for the imposed defocus by adjusting its axial growth rate and choroidal thickness.^34^^–^^36^ As in different animal models, eyes exposed to a spatially low-pass filtered retinal image using frosted diffusers developed abnormal elongation of the eye, subsequently resulting in myopia.^37^^–^^40^ Nevertheless, nonsignificant axial elongation was found in humans after short-term, full-field-of-view adaptation to diffuse blur induced by Bangerter foils.^41^ The mechanisms that underlie the eye's response to low-contrast stimuli remain unclear. However, there is evidence to suggest that the choroid is actively involved in the emmetropization process by changing its thickness to place the retina on the focal plane of the eye as well as releasing growth factors that ultimately lead to changes in the scleral extracellular matrix, altering eye size and refraction.^42^

The primary objective of this study is to investigate changes in choroidal thickness and axial length following short-term wear of spectacle lenses that scatter light and reduce retinal image contrast in young adults. Any regional choroidal thickness alterations are examined in response to full-field scattering lenses versus periphery-only scattering lens wear. The aim of this research is to provide insights into whether retinal image contrast modulation can influence choroidal thickness and axial length and potentially play a future role in slowing myopic ocular growth.

## Materials and Methods

### Study Participants

The prospective study was approved by the Medical Faculty of the University of Tübingen ethics committee and complied with the Declaration of Helsinki and data protection regulations. Before data collection, all participants signed a written informed consent after receiving an explanation of the study's procedure and potential consequences. The inclusion criteria included self-reported systemic and ocular health, corrected visual acuity of ≤0.1 logarithm of the minimum angle of resolution (logMAR), and age between 18 and 40 years. Participants with astigmatism >2.00 diopter cylinder (DC), anisometropia of >1 diopters (D), and a history of orthokeratology wear or refractive surgery were excluded. The following measurements were conducted to confirm the inclusion criteria: screening for corneal and retinal health was performed using wavefront aberrometry (i.Profiler plus; Carl Zeiss Vision GmbH, Aalen, Germany), biometry (ZEISS IOLMaster 700; Carl Zeiss Meditec AG, Jena, Germany), optical coherence tomography (OCT) (ZEISS PlexElite 9000; Carl Zeiss Meditec Inc., Dublin, CA, USA), and the assessment of subjective refraction and visual acuity (ZEISS VISUSCREEN 500 and ZEISS VISUPHOR 500; Carl Zeiss Vision GmbH). Twenty-three participants aged 19 to 35 years (mean age 25 ± 4 years) completed this study, with a group mean spherical equivalent refractive error of −1.54 ± 1.65 D, best-corrected visual acuity of −0.20 logMAR, and baseline axial length of 23.92 ± 0.93 mm.

### Scattering Lens Conditions

To reduce retinal image contrast, single-vision spectacle lenses incorporating the participant's sphero-cylindrical correction were sandblasted in the designated facility (Sigg Strahltechnik GmbH, Lauchingen, Germany). The effect of the scattering lenses was compared to a clear single-vision distance lens—one design involved a clear 8-mm aperture with the participant's full distance correction with the surrounding peripheral lens areas being sandblasted to produce a light-scattering effect, and the other design was entirely sandblasted, giving a full field of light scattering. Each of these two designs was made with two different amounts of light scatter: a medium level, in which the lenses were sandblasted for 25 seconds, and a high level, in which the lenses were sandblasted for 30 seconds. Glass beads ranging in size from 50 to 105 µm were blasted with a pressure of 6 bar (600 kPa) onto the lens surface, which was positioned 30 cm away in consistent circular motions, and the timing of the sandblasting was controlled with millisecond accuracy.

**Figure 1.**
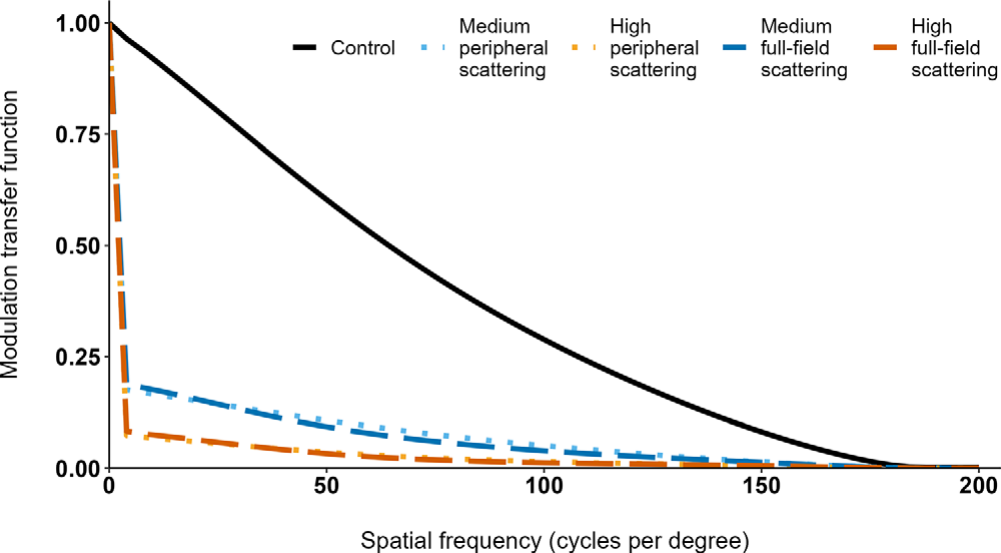
Radial modulation transfer function across the spatial frequencies of 0 to 200 cycles per degree.

The scattering level was determined and aligned through optical characterization, based on spatial light modulation.^32^ Images of the point spread function for each experimental lens condition were captured using a camera unit. For this purpose, a laser source of 532 nm wavelength and an artificial pupil with a diameter of 6 mm were utilized. The radial modulation transfer function of each lens was calculated by performing a fast Fourier transformation of the corresponding point spread function. The characterization involved measurements of lens sets of five participants. These lens sets were chosen with either no sphero-cylindrical lens power or only spherical power, as cylindrical power would have distorted the results. The determined modulation transfer function is depicted in Figure 1.

**Figure 2.**
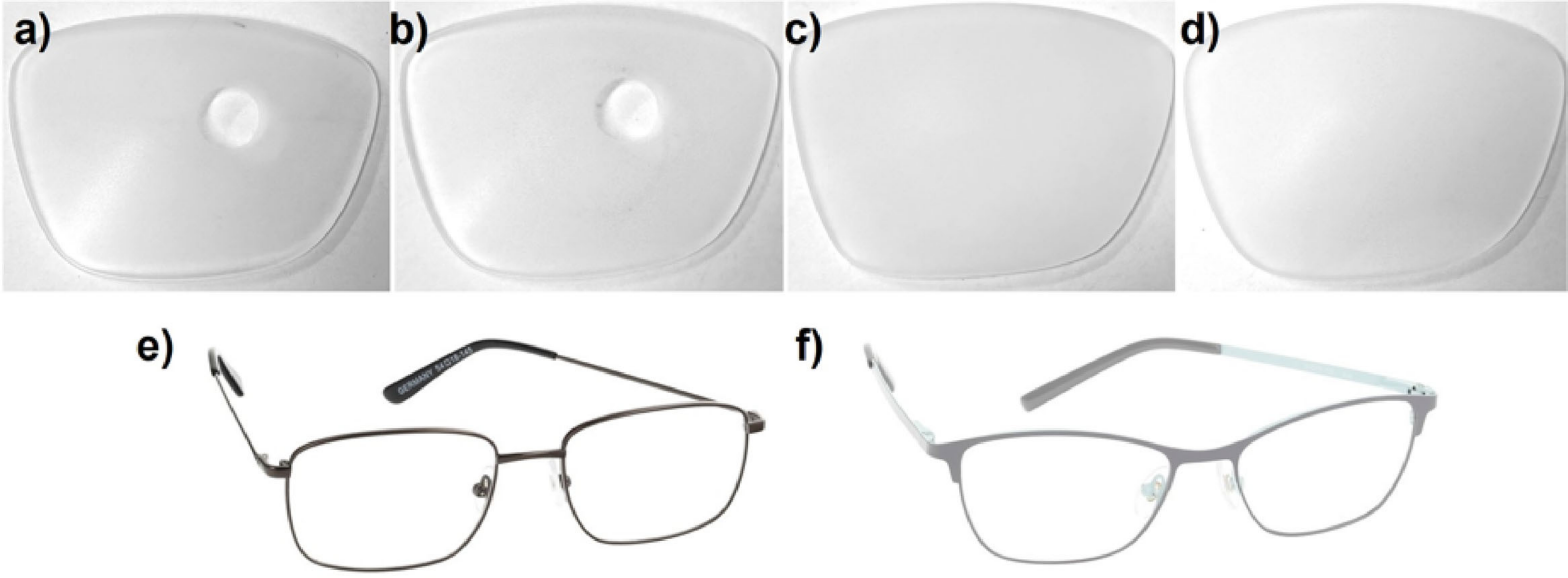
Sandblasted, scattering experimental lens conditions reducing retinal image contrast: (**a**) medium peripheral scattering, (**b**) high peripheral scattering, (**c**) medium full-field scattering, and (**d**) high full-field scattering and frames to mount the experimental lenses: (**e**) smaller frame and (**f**) larger frame.

The effects of scattering experimental lens conditions were compared with a clear lens control condition. During the experiment, the right eyes were treated with the experimental lenses, while the left eye was covered with a sandblasted single-vision lens without refractive power, with the entire lens surface sandblasted for 1 minute beforehand. Visual acuity in the left eye was tested in all participants, with only light perception being reported. The lenses for both eyes were ground into shape and mounted into a spectacle frame. Before the grinding process, the individual pupil distance and mounting height were measured to place the clear central zone of the peripheral scattering lenses to the optical center (see Figs. 2a–d). The spectacle frame was chosen between a smaller or larger size according to the participant's anatomic characteristics of the head (Figs. 2e–f).

### Study Procedure

The four experimental lens conditions and the control condition were tested on different days in random order at the same time each day (between 8 and 11 AM) to avoid diurnal fluctuations in vision,^43^^,^^44^ axial length, and choroidal thickness.^45^ The order of the experimental lenses to be worn was randomized. The same procedure described in the following was carried out for each lens condition. The room lighting was maintained at 20 lux during the experiment, and all measurements were conducted in the same room. Before any study measurement, the participants underwent a 10-minute rest without performing any task to avoid accommodation and change in heart rate and blood flow.^46^^,^^47^ After the washout phase, baseline measurements of choroidal thickness using OCT (ZEISS PlexElite 9000; Carl Zeiss Meditec Inc.), axial length (ZEISS IOLMaster 700; Carl Zeiss Meditec AG), and letter visual acuity were conducted in the same room. The OCT scans were performed between 1040 nm and 1060 nm, with a frequency of 200 kHz and a pattern of 512 × 512 A- and B-scans covering 12 × 12 mm² at the retina. After assessing the baseline measurements, the participants completed a 60-minute lens wear phase to the respective lens condition. During the lens wear phase, the participants were asked to watch a movie at a 4.5-m distance to avoid near-accommodation. Measurements of choroidal thickness, axial length, and visual acuity were then repeated to investigate changes from baseline. For the post–lens wear OCT scan, tracking and follow-up modes were set to capture the same retinal area as in the baseline scan. The changes in choroidal thickness, axial length, and visual acuity after lens wear were subsequently calculated based on the condition- and day-dependent baseline. All measurements were carried out on the treated right eye. The lens was removed for choroidal thickness and axial length measurements but remained in place during visual acuity testing.

### Data Computing

**Figure 3.**
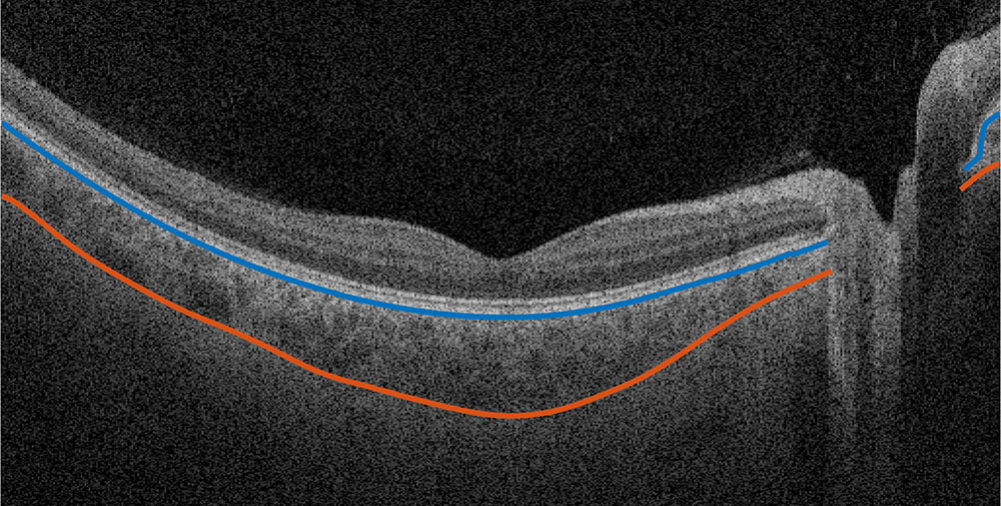
Multilayer segmentation of the choroid (Bruch's membrane in *blue* and choroido-scleral junction in *red*).

**Figure 4.**
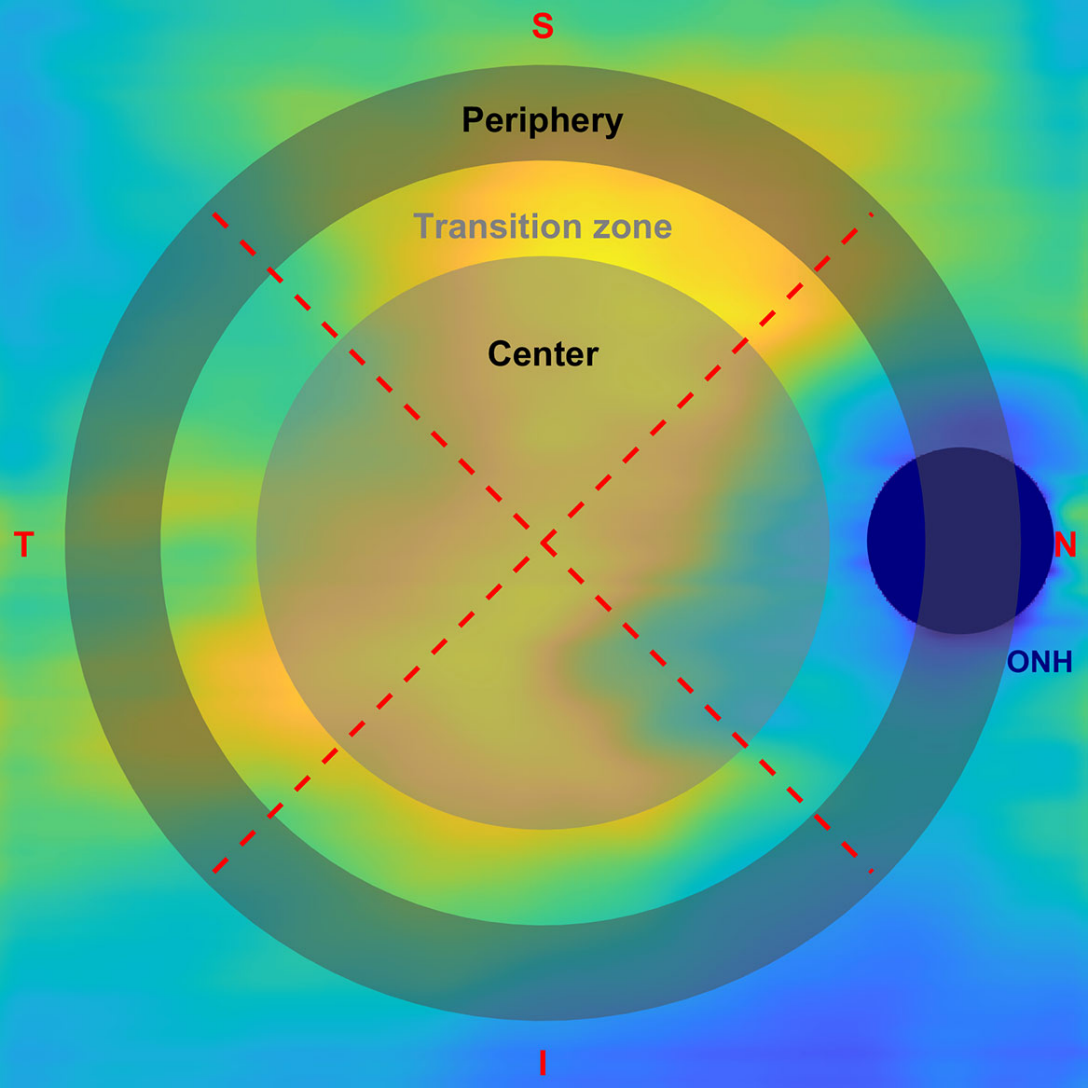
Central and peripheral masks to define retinal areas to analyze choroidal thickness, subsequently subdivided into four quadrants: nasal (N), temporal (T), superior (S), and inferior (I), excluding the area of the optic nerve head (ONH).

Axial length and visual acuity values were derived from the device used. Choroidal thickness was calculated from the OCT scans as follows: Bruch's membrane and choroido-scleral junction were segmented using a validated automated multilayer segmentation from the Advanced Research and Imaging Network (Carl Zeiss Meditec Inc.).^48^ The utilized segmentation software reported a strong correlation and good agreement with manual segmentation of the choroid. The automatically generated segmentation was then manually corrected using MATLAB (version: 9.12.0, R2022a; The MathWorks Inc., Natick, MA, USA; see Fig. 3^4^). The area of the optic nerve head was excluded from further processing. Based on the translation of the experimental lens’ clear zone of 8 mm in diameter onto the retinal level, angular calculations were performed to determine the central (within the clear central zone) and peripheral (outside of the clear zone) retinal areas to be analyzed regarding the choroidal thickness. Therefore, the visual angle of the clear aperture in the nodal plane was calculated from the distance of the back vertex of the lens (standardized at 12 mm for all participants) to the nodal point and then projected onto the retina. The clear aperture covered a viewing angle of 23° on the retinal level. The viewing angle of the clear zone was then transformed into pixel values, resulting in 298 pixels in diameter. This zone is defined as the central zone, while the area outside is referred to as the peripheral zone. To ensure a well-defined clear zone on the retinal level, a transition zone between the clear central zone and the peripheral zone was defined. Subsequently, the diameter of the central retinal area was set to a visual angle of 21° and the inner diameter of the peripheral retina to 28°. The peripheral retinal area was further defined as a ring section and adjusted for its outer diameter to keep an equal number of data points as the central area and thus set to a 39° viewing angle. The central and peripheral zones were subsequently subdivided into four quadrants: nasal, temporal, superior and inferior, see Figure 4.

### Repeatability of Choroidal Thickness Results

The intraoperator repeatability of central choroidal thickness segmentation was 4.75 *±* 13.18 µm, and the intraclass correlation coefficient (ICC) was 0.99 with a mean difference of 1.40 ± 6.74 µm (95% confidence interval [CI], −1.67 to 4.46 µm). Peripheral choroidal thickness analysis reported an intraoperator repeatability of 4.20 ± 11.64 µm, an ICC of 0.99, and a mean difference of 1.86 ± 5.78 µm (95% CI, −0.77 to 4.49 µm). Test and retest axial length measurements reported an intraoperator repeatability of 7.10 ± 9.70 µm and an ICC of 0.99, with a mean difference of 4.46 ± 8.84 µm (95% CI, 0.64–8.28 µm).

### Statistical Analysis

Data were analyzed using R/RStudio (version 2022.07.2; RStudio Team, PBC, Boston, MA, USA) and MATLAB (version 2022a; The MathWorks Inc., Natick, Massachusetts, USA). Visual acuity results are presented as mean and standard deviation. Data for axial length and choroidal thickness are reported as median ± interquartile range (IQR) since normal distribution was refuted by the Lilliefors test. Subsequently, data underwent outlier detection by removing data points located beyond 1.5 IQRs from the median. Statistical analysis was then conducted using a linear mixed model and the post hoc test of estimated marginal means. The analyzed model is defined by either central or peripheral choroidal thickness, central axial length or central visual acuity as the dependent variable, participants as a random effect, and two fixed effects: condition (five levels: control, medium peripheral scattering, high peripheral scattering, medium full-field scattering, and high full-field scattering) and time (two levels: pre– and post–lens wear). The choroidal thickness evaluation included the analysis of chorioretinal locations by eccentricity (two levels: central and peripheral) and quadrant (four levels: nasal, temporal, superior, and inferior). Furthermore, changes in choroidal thickness, axial length, and visual acuity were analyzed by calculating the differences between post– and pre–lens wear values. Subsequently, the linear mixed model was specified by the mentioned change (dependent variable), participants (random effect), and the experimental lens condition (fixed effect).

## Results

### Choroidal Thickness

**Figure 5.**
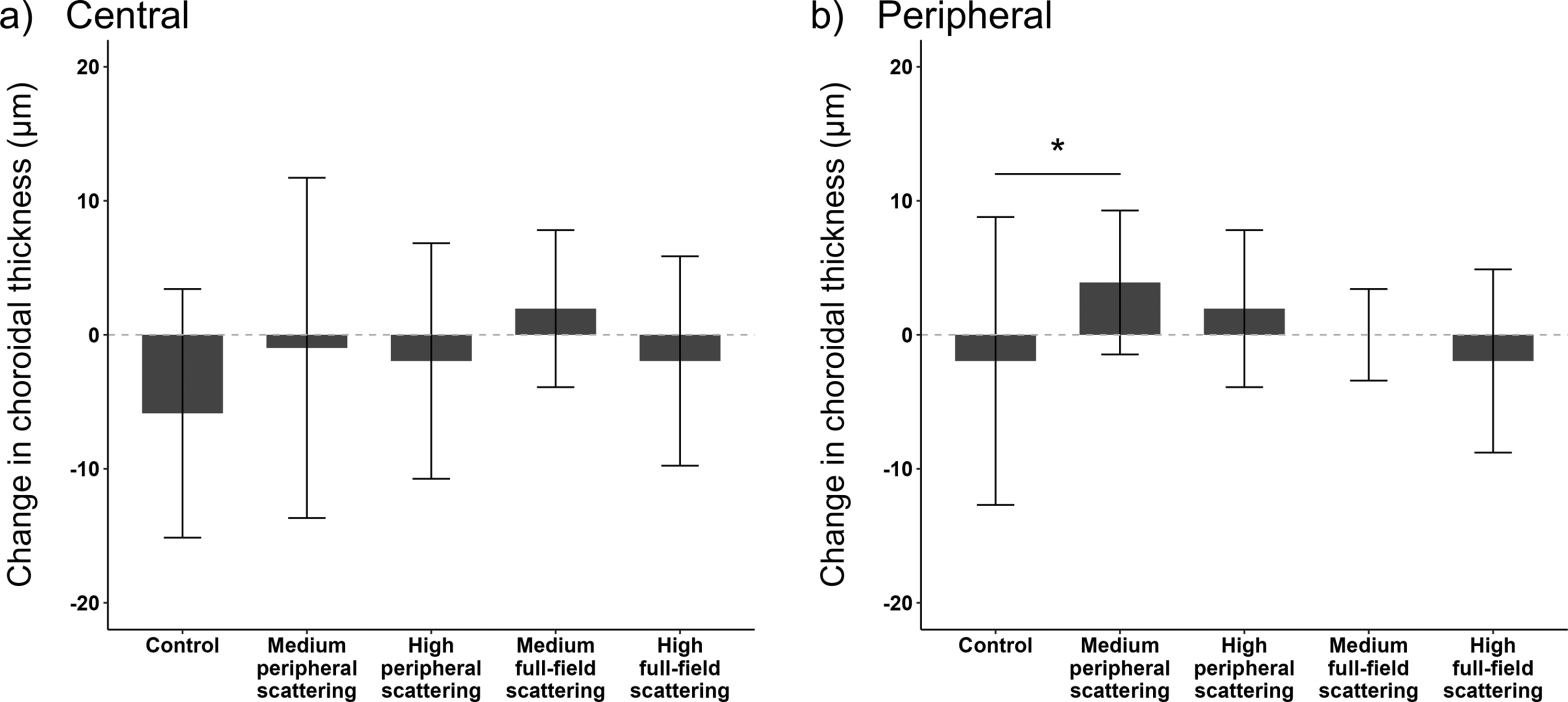
Central and peripheral choroidal thickness (in µm) pre– and post–lens wear of the experimental lens conditions (*n* = 23 participants).

There was no significant effect found either between pre– and post–lens wear on central choroidal thickness, and no significant differences were exhibited among the lens conditions (all *P* > 0.05; see Fig. 5). Central choroidal thickness decreased; however, this change was not statistically significant for any of the test lens conditions, except for the medium full-field scattering condition, which showed an absolute, nonsignificant choroidal thickening on average compared at 60 minutes (+1.95 ± 5.86 µm, *P* = 0.21). The highest reduction of central choroidal thickness was observed in the control condition (−5.86 ± 9.28 µm, *P* = 0.07), followed by the high peripheral scattering condition (−1.95 ± 12.70 µm, *P* = 0.10) and high full-field scattering condition (−1.95 ± 7.81 µm, *P* = 0.06). The least reduction was noted in the medium peripheral scattering condition (−0.98 ± 12.70 µm, *P* = 0.93).

Statistical analysis revealed a significant increase in peripheral choroidal thickness after lens wear to the medium peripheral scattering condition (+3.91 ± 5.37 µm, *P* = 0.04; see Fig. 5). Peripheral choroidal thickness increased nonsignificantly following short-term exposure to high peripheral scattering (+1.95 ± 5.86 µm, *P* = 0.21) and medium full-field scattering (+0.01 ± 3.41 µm, *P* = 0.99). A not significant decrease was found in peripheral choroidal thickness following lens wear to control (−1.95 ± 10.74 µm, *P* = 0.15) and high full-field scattering (−1.95 ± 6.84 µm, *P* = 0.58). When comparing choroidal thickness changes after exposure to experimental lens conditions, the thickness of the peripheral choroid differed significantly between control and medium peripheral scattering (*P* = 0.004).

The quadrant-specific changes in the central and peripheral choroid following lens wear are shown in Table 1. A significant effect was exhibited between the medium full-field scattering and the control condition at the central nasal part (*P* = 0.001). In the superior periphery, a significant difference between the medium peripheral scattering and control condition (*P* = 0.02) was exhibited. No further effects were found between the retinal areas and experimental lens conditions (all *P* > 0.05).

**Table 1.** Nasal, Temporal, Superior, and Inferior Central and Peripheral Choroidal Thickness Changes (Median ± IQR) After Lens Wear to the Clear Control Condition, Medium Peripheral Scattering, High Peripheral Scattering, Medium Full-Field Scattering, and High Full-Field Scattering (*n* = 23 Participants)

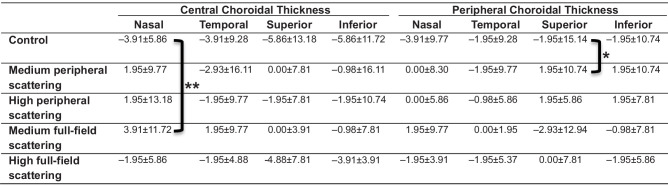								

* *P* < 0.05.

** *P* < 0.01.

### Axial Length

The axial length (see Table 2) did not reveal significant changes after wearing the given experimental lens, and no variations were observed among the different lens conditions (all *P* > 0.05).

**Table 2.** Mean and Standard Error of Foveal Axial Length Changes Before and After Lens Wear of the Experimental Lens Conditions Control, Medium Peripheral Scattering, High Peripheral Scattering, Medium Full-Field Scattering, and High Full-Field Scattering (*n* = 23 Participants)

	Control	Medium Peripheral Scattering	High Peripheral Scattering	Medium Full-Field Scattering	High Full-Field Scattering
Axial length changes (µm)	+1.29 ± 0.47	−5.48 ± 0.72	−0.66 ± 0.69	−4.93 ± 0.68	+1.97 ± 0.54

### Visual Acuity

Foveal visual acuity was measured each time with the experimental lens in place, through the optical center of the lens. Baseline (before the lens wear phase) visual acuity was −0.16 ± 0.06 logMAR for control, −0.16 ± 0.06 logMAR for medium peripheral scattering, −0.17 ± 0.05 logMAR for high peripheral scattering, +0.03 ± 0.12 logMAR for medium full-field scattering, and +0.13 ± 0.10 logMAR for high full-field scattering. Visual acuity was noticeably reduced for the full-field scattering conditions. After the lens wear phase, no significant change was found in visual acuity: 0.00 ± 0.02 logMAR for control, 0.00 ± 0.05 logMAR for medium peripheral scattering, +0.01 ± 0.04 logMAR for high peripheral scattering, +0.04 ± 0.06 logMAR for medium full-field scattering, and +0.03 ± 0.08 logMAR for high full-field scattering. There was no improvement in visual acuity after 60 minutes of lens wear (all *P* > 0.05).

## Discussion

The study investigated changes in central and peripheral choroidal thickness, axial length, and visual acuity after short-term lens wear, of peripheral and full-field experimental scatter lenses across medium and high levels. Peripheral choroidal thickness significantly increased after lens wear of the medium peripheral scattering condition maintaining clear central vision, while the change was significantly increased compared to the control condition. The analysis divided into quadrants showed a main increase in superior peripheral choroidal thickness after exposure to medium peripheral scattering compared to the control condition, while the central nasal choroid was significantly thicker for the medium full-field scattering condition compared to the control condition. The clear lens control condition, as well as the high peripheral and full-field scattering conditions, did not show any significant changes in the central or peripheral choroidal thickness. Choroidal thickness results were compared within each quadrant and not between quadrants, as the latter would not be meaningful and could be misleading. This is because previous literature has reported an asymmetry in nasal and temporal choroidal thickness.^49^^,^^50^ The choroid is nasally thinner than temporally due to the presence of the choroidal watershed area in the nasal region.^49^^,^^50^

Prior research in animal models has explored compensatory choroidal changes in response to optical defocus, resulting in choroidal thickening by wearing positive lenses and choroidal thinning when negative lenses or diffusers were worn.^36^^,^^51^^–^^54^ In humans, short-term adaptation to myopic (positive lenses) and hyperopic defocus (negative lenses) produces results similar to those observed in animal models.^55^^–^^57^ However, short-term choroidal thickness changes in response to diffusers or scattering lenses have not yet been investigated; this study is the first to explore this. Several studies have identified a correlation between choroidal thickness and refractive error, indicating that the choroid tends to be thinner in individuals with higher degrees of myopia.^58^^–^^60^ Additionally, the relationship between choroidal thickness and axial length has been explored, revealing that longer eyes typically exhibit a thinner choroid.^61^^–^^64^ These valuable insights have suggested that variations in the choroidal thickness are associated with future growth rates, making it a significant biomarker for predicting the efficacy of myopia control strategies. Changes in choroidal thickness occur rapidly and prior to long-term changes in axial length.^42^^,^^52^ Therefore, choroidal thickness can be considered an appropriate indicator to assess how the eye responds to visual stimuli. Short-term effects of diffused or scattering lenses on the human choroid and its regional changes have not yet been investigated. However, other investigations of choroidal thickness in response to visual stimuli revealed more prominent changes in choroidal thickness than in the present study.^3^^,^^65^ Reading black text on a white background for 1 hour and vice versa resulted in changes of 16 µm and 10 µm, respectively.^3^ A 60-minute adaptation to +3 D defocus produced regional choroidal thickness changes up to 7 µm.^65^ Previous studies determining choroidal thickness with ZEISS PlexElite, which has been used in the current study, found similar or smaller changes in choroidal thickness in response to visual stimuli as in the present study. One study investigated choroidal thickness responses to 30-minute wear of multifocal contact lenses of +2.5 D and found changes of 2 µm.^66^ Another study examined 20-minute light stimulation on choroidal thickness and found changes of 3 µm.^67^

In the current study, a significant difference in the peripheral choroidal thickness was found after lens wear of the medium peripheral scattering condition (+3.91 ± 5.37 µm, *P* = 0.04) compared to the control condition (−1.95 ± 10.74 µm), with a rounded difference of 6 µm (*P* = 0.004). A stronger reduction in retinal image contrast by exposure to the high peripheral scattering condition did result in an absolute increase in peripheral choroidal thickness (+1.95 ± 5.86 µm) but was not a significant effect (*P* = 0.21). It is assumed that the retina can adjust the choroidal thickness by altering the blood flow based on the amount of peripheral contrast reduction.^68^ The retinal ON and OFF channels play a crucial role in retinal contrast signaling. It is assumed that the activity of both channels is altered when image contrast decreases. Additionally, it is suggested that these ON and OFF channels, specifically their receptive fields, transmit a signal to the choroid to adjust its thickness.^69^

Lens wear of medium or high peripheral scattering lenses with clear apertures did not affect central choroidal thickness (*P* > 0.05). The obtained results highlight a localized regulatory mechanism of the choroidal thickness in response to visual stimuli, consistent with previous findings in both human^65^ and animal studies.^51^^,^^70^^–^^75^ These findings align with research suggesting that variations in peripheral image quality have a considerable impact on refractive development.^27^^,^^69^^,^^76^^,^^77^ A possible underlying explanation is that the absolute number of neurons in the periphery is greater than in the fovea, and therefore, the signals of the retinal periphery might overlay foveal signals, affecting general ocular growth.^27^^,^^28^ Another reason could be that selective, critical elements in the signal cascade regulating axial elongation are distributed in the periphery.^28^

Medium and high full-field scattering conditions did not change general central or peripheral choroidal thickness following short-term lens wear (all *P* > 0.05). Consequently, it might be beneficial to maintain a clear central aperture, providing the participants’ comfort and compliance when wearing and, at the same time, increasing choroidal thickness in the periphery. Breher et al.^66^ found nonsignificant choroidal thickening at the nasal part after 30 minutes of multifocal contact lens wear. In the current study, changes in the nasal part were found as well after 60 minutes of wear of the medium full-field scattering condition, suggesting that the nasal central retina might be able to detect changes in image contrast compared to superior, inferior, and temporal. Another study investigated the influence of hemifield defocus on superior and inferior choroidal thickness after 60 minutes of lens wear and exhibited an increased superior choroidal thickness after superior exposure without influencing the inferior choroidal thickness and vice versa.^65^ However, greater changes in choroidal thickening were found in the superior retina.^65^ In the current study, changes in the peripheral superior choroidal thickness were found after lens wear of the medium peripheral scattering condition. The previous and current findings suggest a pattern of regional sensitivity in the retina to changes by image contrast. This study extends existing findings by investigating choroidal thickness responses to lens wear of medium and high peripheral and full-field scattering conditions. This supports the idea that different retinal regions have distinct responses to visual stimuli, such as scattering, with the peripheral superior retina and nasal central retina showing significant differences in medium full-field scattering and medium peripheral scattering, respectively.

In comparison to the scattered lens conditions, the clear lens control condition did not degrade retinal image quality, but an absolute, nonsignificant thinning of the choroid was found in the center (−5.86 ± 9.28 µm) and periphery (−1.95 ± 10.74 µm). The control condition was included in the study design to elaborate changes in choroidal thickness, which cannot be attributed to a visual stimulus (e.g., diurnal fluctuations, blood pressure, and hydration status, all of which can influence the choroidal blood flow and therefore choroidal thickness). The slight, nonsignificant choroidal thinning observed following exposure to the control condition is likely due to residual diurnal variation^45^^,^^46^ or dim room lighting.^78^^,^^79^ Previous literature has documented similar slight choroidal thinning occurring between 9 and 11 AM.^45^ As the choroid undergoes diurnal variations, all measurements were conducted at the same daytime to eliminate diurnal effects on this study's investigations. Moreover, we conducted baseline measurements before each experimental lens wear phase to keep the fluctuations small and calculated the postadaptation change from the respective condition-dependent baseline. However, due to time constraints and participant availability, it was not possible to keep the number of rest days constant for each participant.

It is worth mentioning that all scattered lens conditions did not reach the amount of central choroidal thinning after lens wear to the clear control condition. As previously shown, visual-induced choroidal changes impact the diurnal rhythm of thickness fluctuations.^80^^–^^82^ This study could not prove a complete diurnal rhythm impact in exposure to scattering and needs further investigation. Possible confounders related to participant positioning and eye movement during the imaging process were controlled using the tracking mode of the OCT imaging system and therefore can be ruled out. To identify changes in choroidal thickness after exposure to scattering different from the control condition, a pairwise analysis against the control condition was performed. However, the OCT system's capability must be considered in terms of the effect size. While the OCT demonstrated a repeatability of 4 µm and thus falls below the effect size between the control condition and medium scattering condition, the repeatability of the IOLMaster for axial length was 7 µm. Given the resolution limitations of this biometer, accurately assessing small axial length responses is challenging, which led to nonsignificant variations observed in the current study.

A human study observed an elongation in axial length following short-term full-field-of-view exposure (10, 20, and 30 minutes) to a high-level scattering condition using the Bangerter foil of density 0.2.^41^ Significant changes in axial length were not found in the current study. However, the level of scattering was not as high as used by Teoh et al.,^41^ where the image degradation in terms of visual acuity was 0.8 logMAR. This study reached a maximum degradation in terms of visual acuity of 0.5 logMAR for the high full-field scattering condition. Therefore, the results of this study cannot draw any definitive conclusions regarding the relationship between axial length and choroidal thickness, as the axial length was measured at the fovea while macular choroidal thickness was assessed with a wider field of view. Another study shows axial elongation after short-term exposure to calculated low-pass filtered movies. However, when matching the contrast modulation of calculated low-pass filtered movies and real positive defocus of +2.5 D, the latter led to axial shortening in emmetropes. In myopes, no differentiation between real and calculated defocus was found as both conditions led to axial elongation.^83^ Subsequently, previous literature results suggest that myopes have a reduced ability to distinguish between various strategies to reduce retinal image contrast.

Further previous studies reported enhanced visual acuity following both long-term (6 and 12 weeks) and short-term exposure (40 minutes) to full-field scattering induced by Bangerter foils.^84^^,^^85^ The full-field scattering conditions reduced visual acuity in the current study, while the control condition and the peripheral scattering conditions did not affect visual acuity. Visual acuity remained the same for the respective conditions before and after the lens wear phase. An adaptation effect in visual acuity was not found in the current study after full-field lens wear or peripheral-only exposure to scattering (all *P* > 0.05). Other visual performance assessments found a decrease in contrast sensitivity after short-term adaptation (30 and 90 minutes) to peripherally induced scattering by Bangerter foils. ^86^

The literature shows different time durations for adapting to scattered lenses, extending from minutes to weeks.^84^^–^^87^ In general, contrast adaptation processes are to be distinguished into short-term changes and long-term changes. Both mechanisms are based on a form of cortical neuroplasticity, recalibration of the visual response to compensate or boost for variations in spatial contrast.^85^^,^^88^ The compensation and boost signal process and its relation to choroidal thickness modulation need further investigation. Regarding the choroid, visually induced differences in its thickness have been observed previously in the literature after minutes^89^ to an hour.^56^^,^^57^^,^^65^^,^^81^^,^^90^^–^^92^ Furthermore, it is crucial to explore the long-term effects of reduced contrast in the peripheral retinal image on both central and peripheral choroidal regions. This assessment will provide clarity on the potential of short-term choroidal effects as predictors of long-term consequences.

Scattered lenses reduce contrast over all spatial frequencies, thereby affecting vision at any time. Previous studies found selective channel activation affects the choroidal thickness. It was discovered that ON channel activation increases the thickness of the choroid, while OFF channel stimulation results in choroidal thinning.^3^^,^^93^ The ON and OFF channel receptive fields, structured in a center-surround system, might be concatenated by subsets and therefore be able to respond locally.^65^

This study observed that the peripheral retinal area exhibited choroidal thickening after exposure to medium peripheral scattering. Conversely, the central retinal area did not show a response in choroidal thickness. Additionally, it is noteworthy that only a specific peripheral scattering condition influenced peripheral choroidal thickness in our study, as the high peripheral scattering condition did not affect choroidal thickness. The central choroidal thickness was also only influenced nasally by the medium full-field scattering condition and not by the high full-field scattering condition. This suggests that the regional exposure and the degree of reduction in retinal image contrast may play a crucial role in the subsequent signaling cascade. Based on the regional changes, it could be assumed that only the periphery is affected by peripheral scattering and that the center is favored over the periphery by full-field scattering. However, more research is needed to give a clear answer. Regarding the degree of scattering, there is no literature available that specifically addresses the influence of different levels of scattering on human choroidal thickness. Therefore, the reason for the current study's finding is unknown, whereas it can be speculated that a determined level of scattering is needed to trigger changes in choroidal thickness. Still, short-term adaptation in dependence on axial length changes has been studied. In a previous study, 10- to 30-minute adaptation to strong diffusers, such as light-perception Bangerter foils or Bangerter foils of density 0.2, led to axial elongation.^41^ Considering that an increase in axial length is related to choroidal thinning, a high level of scattering might be suggested to promote rather than counteract myopia progression. Moreover, the current study cannot provide information on whether the ON or OFF channel was activated concerning choroidal thickening. In a previous study, the psychophysical assessment of ON and OFF receptive field processing showed differences in contrast sensitivity between emmetropes and myopes, but it was not affected by contrast reduction induced by Bangerter foils.^94^ Previous and current findings do not shed light on the mechanism by which contrast reduction prevents the development of myopia.

An essential aspect of understanding changes in contrast produced by each experimental lens condition lies in the analysis of the radial modulation transfer function (see Fig. 1). When comparing the modulation transfer function of the medium scattering lens condition used in this study with the diffusion optics technology lenses, previously reported in the literature,^95^ it becomes apparent that contrast modulation occurs similarly for spatial frequencies above 40 cycles per degree but differs for lower spatial frequencies. This difference can likely be attributed to variations in lens design and manufacturing processes. The significant effects found in the current study are relatively small. This could be attributed to the contrast modulation of the experimental lens design across different spatial frequencies, as well as the short duration of lens wear. Due to the contrast modulation created by the lens design, it might be essential that low spatial frequencies are not affected or that very low and middle or high spatial frequencies differentiate not too strongly. It is also worth noting that any long-term effects cannot be explained and transferred by the results of this study.

In animal studies, exposure to scatter lenses led to myopia development.^28^^,^^96^^–^^99^ Ambient lighting is another crucial factor that influences the scattering affecting retinal image quality and, therefore, must be considered in our results. Previous optical evaluations have shown that intense light conditions increase scattering, significantly reducing contrast in the retina.^32^ Consequently, ambient light levels fluctuate throughout the day, while research studies are typically conducted under constant lighting conditions. Furthermore, the pupil size affects the degradation of the retinal image by induction of diffused lenses. In the current study, pupil size was not controlled, as the intention was to replicate natural conditions. The same applies to eye and head movements. Moreover, natural accommodation was also not controlled. However, participants were instructed to watch a movie at a far distance during the adaptation period to avoid near-accommodation. Previous literature found axial elongation and choroidal thinning linked to accommodation.^47^ However, the present study did not reveal a significant modulation in axial length, and no thinning of the choroid was observed. The level of scattering accompanied by contrast reduction, lens design, and exposure duration are, however, crucial factors in predicting the potential success of slowing down myopia progression.

## Conclusions

Peripheral choroidal thickening was found after short-term lens wear of the medium peripheral scattering condition, most expressed in the superior peripheral retina, compared to the control condition. Nasal central thickening was explored after exposure to the medium full-field scattering condition in comparison to control. Neither central nor peripheral choroidal thickness was affected by the high peripheral scattering condition or high full-field scattering lens condition. The results indicate local retinal contrast detection mechanisms that signal the choroid to thicken. This finding gives insight into the mechanism behind the myopia control strategy using peripheral scattering and the importance of the peripheral retina in myopia research. Further research is required to evaluate choroidal thickness changes after exposure to scattering lenses concerning the lens design, level of contrast reduction, lens wear time, lighting conditions, ON and OFF channel activation, and long-term effects on refractive development.
